# Classification of audio signals using spectrogram surfaces and extrinsic distortion measures

**DOI:** 10.1186/s13634-022-00933-9

**Published:** 2022-10-22

**Authors:** Jeremy Levy, Alexander Naitsat, Yehoshua Y. Zeevi

**Affiliations:** 1 Faculty of Electrical Engineering, Technion, Haifa, Israel; 2 Faculty of Biomedical Engineering, Technion, Haifa, Israel

**Keywords:** Distortions measure, 1D signal processing, Classification, Spectrogram embedding, Surfaces, Manifold, Geometric feature engineering

## Abstract

Representation of one-dimensional (1D) signals as surfaces and higher-dimensional manifolds reveals geometric structures that can enhance assessment of signal similarity and classification of large sets of signals. Motivated by this observation, we propose a novel robust algorithm for extraction of geometric features, by mapping the obtained geometric objects into a reference domain. This yields a set of highly descriptive features that are instrumental in feature engineering and in analysis of 1D signals. Two examples illustrate applications of our approach to well-structured audio signals: Lung sounds were chosen because of the interest in respiratory pathologies caused by the coronavirus and environmental conditions; accent detection was selected as a challenging speech analysis problem. Our approach outperformed baseline models under all measured metrics. It can be further extended by considering higher-dimensional distortion measures. We provide access to the code for those who are interested in other applications and different setups (Code: https://github.com/jeremy-levy/Classification-of-audio-signals-using-spectrogram-surfaces-and-extrinsic-distortion-measures).

**Supplementary Information:**

The online version contains supplementary material available at 10.1186/s13634-022-00933-9.

## Introduction

Surfaces and higher-dimensional manifolds can be endowed with extrinsic, well-defined, geometric structures. Utilizing properties of surface geometry can be advantageous in the development of algorithms that involve mappings for the purpose of assessment of similarities by means of meaningful geometry-based distortion measures, and in their efficient computations [1, 2]. We have previously presented a complete framework of this approach, along with its practical applications in computer graphics and computer vision [1, 3, 4]. The purpose of our present work is to adopt this powerful approach for the benefit of processing, similarity assessment, feature engineering, and classification of one-dimensional signals, such as pulmonary sounds and speech. Our novel approach to feature engineering that emerges as a by-product of our approach can enrich the representation of signals that are fed into the classification stage of a machine learning architecture and thereby enhance the merit of application of machine learning in signal processing and classification. To take advantage of our approach, and benefit from the rich geometric feature space that characterizes a surface, we have to first embed the one-dimensional signal in, or represent it by a surface. This is accomplished by utilizing one of the handful of transformations of a signal into a combined space [5], such as the time–frequency (i.e., the spectrogram [6]), or any other two-dimensional combined space (e.g., Gaborian, or time scale, i.e., wavelets [7]). In this study, we adopt the spectrogram representation that has been shown repeatedly to be effective in processing, detection, and classification of speech [8] and lung sounds [9], which we use as examples.

We consider the spectrogram to be a geometric object, embedded in a three-dimensional Euclidean space, wherein the *x*- and *y*-axis represent the time and frequency, and the *z*-axis, the so-called instantaneous spectrum. Surfaces are characterized by their geometric properties, such as distances and curvatures. The representation of signals by surfaces allows us, therefore, to extract features that are based on a metric that quantifies geometric distances between surfaces. In the context of signal processing and classification, such geometric properties can enrich the set of features that are used for signal recognition and classification. For example, with reference to our test case of speech, we validate the advantages of utilizing our approach in recognition and classification of speakers’ accents. In the case of pulmonary sounds, we demonstrate how powerful our approach is in classifying patients into equivalence classes of various diseases.

It is of interest to note, in the context of the geometrical structure of the spectrogram representation by a surface, that the curvature corresponds to the bandwidth or ‘local bandwidth’ of a signal represented by the surface [10]. Thus, our approach to one-dimensional signal processing and classification is based on the idea of representing a given signal segment by a unique geometric object. It allows us to combine existing signal processing methodologies and/or classical, ad hoc, feature selection, that is widely used in feature engineering for the purpose of classification by machine learning methodologies and architectures, with geometry-based formalism and its by-product of the well-defined features that have geometrical meaning, elsewhere used in shape analysis, mappings, and classification [11]. To further enhance the strength of our geometric-based feature engineering in the context of sound signals classification, we incorporate and combine the classical features of mel-frequency cepstrum coefficient that are widely used in speech recognition [12–14], with shape descriptors that characterize our geometric objects.

As is the case in computer graphics, computer vision or any computational processing that is applied to manifold surfaces embedded in $R^3$, the first step of the processing is the sampling and triangulation resulting in a triangle mesh (Sect. 2.1 for further details). Given two meshes, representing two surfaces, i.e., geometric objects and, in turn two one-dimensional sound (or other one-dimensional) signal segments, we define a metric, suitable for quantifying the similarity between these two meshes. This is accomplished by optimally deforming one of the meshes onto the other one and assessing the extent of deformation required for this matching process (Sect. 2.2).

Given a large set of geometric objects, i.e., signal segments represented by meshes, which are the discrete versions of the manifold surfaces, it is not practical to estimate the required deformations and corresponding similarities by pairwise comparisons; this would not be computationally feasible. Instead, we map each one of the geometric objects onto a reference, target, domain by a process known as surface flattening, or parametrization [1], and assess in doing so the transitive similarity. Such a target domain (actually a canonical domain [1]) may, for example, be a circle [15]. To execute this non-convex mapping of geometrical data ‘optimally,’ we adopt a recently proposed adaptive block coordinate descent (ABCD) algorithm [2]. The geometric distortions induced by the mapping of the geometric objects are then used as measures of the dissimilarity of the geometric objects and, in turn, of the signal segments that they represent. This is the essence of our clustering into equivalence classes and of the classification.

Among the conceptual and practical contributions of our approach to representation and analysis of 1D signals, we wish to stress already at the outset the importance of the novel interpretation of the spectrogram (or representation of a 1D signal in one of the alternative combined spaces [5]) as a surface embedded in a Euclidean space. Further, based on our empirical evidence, and theoretical considerations to be presented elsewhere, it is asserted that the two-dimensional (2D) surfaces which represent the 1D signals do not self-intersect and constitute 2D manifolds [16]. Therefore, as a consequence of our interpretation, geometric properties of the 2D manifolds can be used to derive a metric for similarity assessment, change detection and quantification and similar tasks. We accomplish these tasks by adopting and modifying our previously presented adaptive block coordinate descent (ABCD) algorithm [2], developed for robust geometric optimization, and by extracting distortion measures from the optimal mappings of the discrete surfaces, i.e., meshes. Whereas in our previous studies we measured distortions on tetrahedral meshes of volumetric domains, for assessing similarities in medical images [11, 17], and for analyzing shapes in computer graphics [4], here we use for the first time distortion measures for 1D signal processing, by representing the 1D signals as meshes of surfaces. In terms of enriching the toolbox available to the signal processing community, our method is the first to fully integrate geometric algorithms and distortion estimation techniques with classical machine learning tools, to obtain an end-to-end framework for classification. Further, our study is the first to perform statistical analysis of distortion features and to quantitatively measure precision of various classifiers that employ distortion features. Previous methods have only demonstrated qualitative results in the form of distortion scatter plots or distortion heatmaps.

Results obtained in the classification of speech accents and of sounds characteristic of lung diseases are presented in Sect. 4. Encouraged by these results, we address in the discussion potential promising extensions of our approach, by considering higher-dimensional distortion measures, for example, sound signals characterized by three-dimensional distortions, induced by tetrahedral meshes that are mapped onto canonical domains. We also address possible extension of our methodology by curvature-based sampling of the surface [10], that is likely to enhance its quality in certain applications.

## Methods

**Fig. 1:**
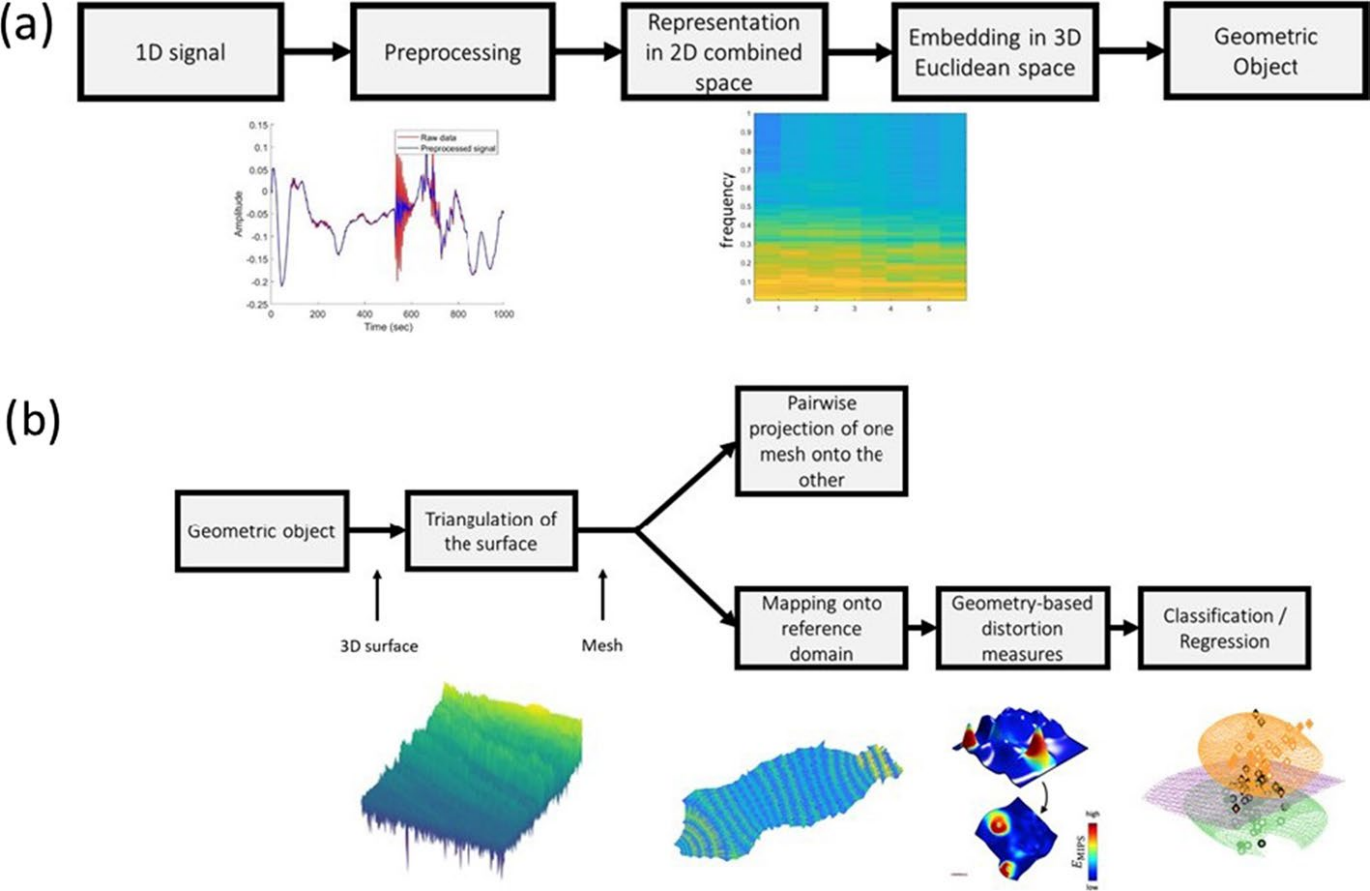
High-level schematic overview of the proposed model. **a** Highlights the ‘embedding’ process. Subsequent to the preprocessing, the spectrogram of the 1D signal is computed and presented as a surface. This allows the conversion of the original problem of 1D signal analysis to geometric analysis of manifolds. The second part of the proposed method begins with this ‘geometric object,’ which is the output of the first part. **b** Summarizes how this geometric object is used for the extraction of features. First, the spectrogram is converted to a mesh, by means of the Delaunay algorithm. Then, the meshes are projected into a target domain by using the ABCD algorithm. The meshes are clustered in this target domain, with reference to a well-defined metric. Finally, distortions measures are computed from the obtained meshes

Figure 1 depicts a high-level schematic overview of the proposed algorithm. The schematic framework of our approach is divided into two major parts. The first one (Panel (a)) is concerned with the representation of the 1D signal by a geometric object. This is the essence of the ‘embedding’ process, i.e., the conversion of the one-dimensional signal into a ’geometrical object,’ i.e., a surface. Subsequent to the preprocessing that combines straightforward conditioning and denoising, the spectrogram is computed and represented as a surface embedded in a three-dimensional Euclidean space. The original problem of conventional 1D signal analysis and classification is thereby converted to geometric analysis of surfaces or higher-dimensional manifolds.

The second part (Panel (b) of Fig. 1) highlights how is the geometric object used in the extraction of highly descriptive geometric features and how the latter are implemented eventually in pairwise similarity assessment (upper branch) or signal classification lower branch. To this end, the 3D spectrogram has to be presented by its discrete surface, which is obtained by implementing an algorithm of triangulation. [Note that this discretization process results in a non-uniform discrete representation of the surface.] The obtained mesh is then deformed onto another mesh for assessment of their similarity (top branch) or, alternatively, mapped into a reference domain (bottom branch), wherein all inter-distances between all the meshes corresponding to the 1D signals are clustered with reference to a well-defined metric. Utilizing the proposed approach in the context of machine learning, surface distortion measures extracted in the process of mapping onto the reference domain become available for capability of feature engineering utilized in the design of the machine learning architecture.

The first two stages are aimed at computing a discrete geometric representation of the signals, whereas the goal of the last two stages is to compare these discrete representations. The technical details of the above stages of the algorithm are presented in the sequel.

### Sampling and triangulation

Assume that *S* is a manifold surface, embedded in $\mathbb {R}^3$, and that $\mathcal {V}$ is a finite set of points (vertices) sampled on *S*. Then, a common way to discretize *S* is to divide it into a finite set of triangles $\Im$ such that: (**i**) vertices of the obtained triangles belong to $\mathcal {V}$; $({\textbf {ii}})$ for any pair of non-disjoint triangles $t_1,t_2\in \Im$ the intersection $t_1 \cap t_2$ is either a common edge of $t_1$ and $t_2$ or a common vertex of these triangles. We will refer to the pair $(\mathcal {V}, \Im )$ as to the triangle mesh of a surface *S*.

In our case, each input signal *I* is represented by a spectrogram surface $S=S(I)$ that can be written in the following parametric form:

1 $$\begin{aligned} S(I)=\big \{\big (x,y,z(x,y)\big ) | x\in X, y\in Y \big \}\,, \end{aligned}$$

where *X* and *Y* are the time and frequency ranges of the signal *I*. We divide *X* and *Y* into a number of uniformly distributed points $x_1,\ldots ,x_N$ and $y_1,\ldots ,y_N$, and the vertex set $\mathcal {V}$ of *S* is defined by

2 $$\begin{aligned} \mathcal {V} = \big \{(x_i,y_j, z(x_i,y_j))|\, i,j = 1,2,\dots ,N \big \} \,. \end{aligned}$$

However, in some scenarios, using adaptive sampling can potentially yield even better results. (See Sect. 5 for the discussion on more advanced sampling schemes.)

The triangle set $\Im$ of *S* is constructed by the standard algorithm of Delaunay triangulation [18] that minimizes the minimal angles in all of the triangles in $\Im$. This triangulation algorithm avoids generation of slim triangles whose appearance may lead to numerical issues at the stage of the feature extraction.

Subsequent to representing data by triangular meshes, we proceed to the next step of analyzing geometric properties of these meshes.

### Shape descriptors

Given two meshes of spectrogram surfaces, we wish to define a metric suitable for quantifying geometrical similarities between these meshes. [In computer vision, such metrics are often referred to as *shape descriptors*.] We use here the deformation-based method. In such methods, a distance between two shapes $S_1$ and $S_2$ is estimated by computing an optimal deformation $f_{12}$ of $S_1$ while projecting it onto $S_2$, and by measuring changes in various geometric features induced by $f_{12}$. There exist many criteria for definition of map’s optimality. Most of these criteria are targeted at preserving the map injectivity and avoiding visual distortions, as much as possible.

Note that for a large collection $\{S_1,\ldots ,S_m\}$ of shapes it may be very demanding to compute optimal deformations $f_{ij}$, for each $1 \le i<j\le m$. Therefore, instead of matching all the pairs of shapes, a more practical approach is to compute an optimal mapping $f_i$ of each shape $S_i$ into a simple target domain. Such a target domain (actually a canonical domain [1]) may, for example, be a sphere [19], a circle [15], or a plane. In the two examples shown in this paper, our source domains are spectrogram surfaces. Since these surfaces have a disk topology, we map them into a plane by a process known as surface flattening, or parametrization [1].

Our model employs deformation-based descriptors for measuring similarities between triangular meshes. Note that all the meshes that constitute a peak surface of spectrograms have the topology of a planar disk. Therefore, a natural candidate for the optimal deformation of such a mesh *M* is a length-minimizing mapping of *M* into the plane. We refer to this mapping process as to the *surface flattening*, for short. In our model, surface flattening algorithms are used for computing deformation-based descriptors of spectrographic shapes.

If *f* is a flattening of a mesh *M*, we select the shape descriptors of *M* to be the geometrical distortions that measure how Euclidean lengths are deformed under *f*. In such a case, each mesh *M* can be associated with its signature vector $(E_1,\ldots E_2)$, where numbers $E_i$ are various estimates of the metric deviations induced by flattening *M* into the plane.

In the sequel, we address in detail the surface flattening and the distortion estimation processes.

### Surface flattening

Surface parametrization tasks can be reduced to the following optimization problem:

3 $$\begin{aligned} \begin{aligned} f^*=~&\underset{f}{\mathrm{argmin}}~ E(f); \\ \text {s.t. }&\det df_t > 0, t\in \Im \,, \end{aligned} \end{aligned}$$

where $f^*$ is a piecewise affine mapping of a mesh $(\mathcal {V},\Im )$ that minimizes the chosen distortion criteria *E* under the following constraints: For each mesh triangle *t*, the component of $f^*$ on *t* is an orientation preserving map. These constraints are expressed by the determinant signs of Jacobian matrices $df_t$, $t\in \Im$. Negative determinants of the Jacobians yield inverted triangles in the image of *f*. Satisfying the orientation constraints is therefore the necessary condition for inducing one-to-one parametrization of surface meshes.

We adopt the recently proposed adaptive block coordinate descent algorithm [2] (ABCD), combined with the Tutte embedding method [20], to solve the optimization problem (3) and thereby the parametrization problem. In particular, we initialize the parametrization problem (3) by mapping triangular meshes onto a circle via the method of [20]. We then employ the ABCD algorithm to induce locally injective parametrization characterized by minimal length distortions.

**Fig. 2:**
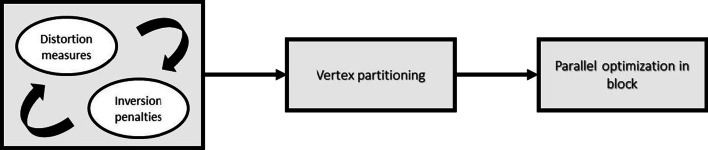
High-level flowchart of the ABCD algorithm. First, a global descend field is applied alternatively on modified distortion measures and inversion penalties. Then, the vertex is partitioned and each block is locally optimized

The ABCD algorithm performs a high-quality mapping of geometrical data, using inversion-free simplicial mappings with low shape distortions. This is done by an alternative optimization process of modified distortion measures (isometric and conformal) and inversion penalties. The algorithm starts with block coordinate descent optimization, which modifies the subset of vertices and converges to global solver. Figure 2 presents a high-level flowchart of the algorithm.

Note that, since (3) is a non-convex problem, solving it with different initial maps may lead to distinct local minima. Therefore, choosing an appropriate initialization method is crucial for adequate approximation of the global minimizer $f^*$.

**Fig. 3:**
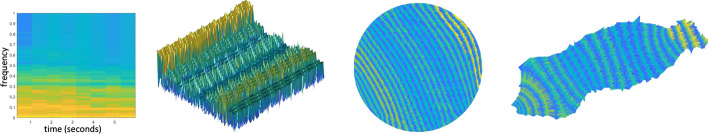
Visualization of the process of measuring distortions associated with the parametrization problem. The figure depicts, from left to right: a sound spectrogram, a triangular mesh $(\mathcal {V},\Im )$ representation of the spectrogram surface, an initial mapping of $(\mathcal {V},\Im )$ onto the plane and the final flattening of the mesh, computed by means of the ABCD algorithm

We tested a number of different initialization schemes and found that using a convex combination mapping of meshes [20] onto a planar disk yields the best results. Note that the algorithm of [20] is actually a variant of the classical Tutte embedding algorithm that is widely used in shape processing applications. This method guarantees a bijective mapping onto convex planar domains, and it has a low computational cost (see Additional file 1: Sect. 7.1). Figure 3 demonstrates this initialization scheme and the related process of distortion minimization.

We proceed to discuss the process of feature extraction. It includes the *local sub-step* of extracting features of individual triangles and the *global sub-step* in which local features are summed over large subsets of mesh triangles.

### Measuring local distortions

If $M=(\mathcal {V},\Im )$ is a triangle mesh and *f* is a simplicial mapping of *M*, then a *local distortion* induced by *f*, on a triangle *t*, is defined to be a function $E(\sigma _1,\sigma _2)$ of the singular values $\sigma _1(df_t)$ and $\sigma _2(df_t)$ of the Jacobian $df_t$.

The Jacobian singular values uniquely define the shape of a triangulated surface, up to rotation and sliding of mesh triangles. Generally speaking, local distortions estimate how extensively is the shape of *t* distorted under *f*.

These measures are instrumental in many applications in computer vision, including shape classification and shape analysis [17, 21]. In our algorithm, geometric distortions are used as measures of dissimilarity of triangulated surfaces.^1^

Note that for a dense triangulation, feeding singular values $\big \{ \sigma _i(df_t)| t \in \Im ,\, i=1,2\big \}$ to a deep learning model preserves all the information contained in the pixels of the spectrogram. Our algorithm employs several distortion measures. These distortions belong to the following major classes of geometric measures:

**Isometric distortions:** These measures estimate distortions of the Euclidean length. We use the following isometric distortions:

- As-Rigid-As-Possible (ARAP) energy [22]
  4 $$\begin{aligned} E_{\mathrm {ARAP}} (\sigma _1, \sigma _2 ) = (\sigma _1^2 -1)^2 + (\sigma _2^2 -1)^2\,; \end{aligned}$$
- Symmetric Dirichlet energy [23]
  5 $$\begin{aligned} E_{\mathrm {SD}} (\sigma _1 , \sigma _2 ) = \displaystyle \frac{1}{4} \, (\sigma _1^2 + \sigma _1^{-2} + \sigma _2^2 + \sigma _2^{-2})\,; \end{aligned}$$
- Quasi-isometric (*qi*) dilatation [4, 24]
  $$\begin{aligned} E_{\mathrm {QI}} (\sigma _1 , \sigma _2 ) = \max \limits \, \{ \sigma _1 , \sigma _2^{-1}\}\,; \end{aligned}$$

**Conformal distortions:** These distortions estimate how far *f* is from being an angle-preserving mapping. Our algorithm uses the following estimates of conformal distortions:

- Quasi-conformal *(qc)* dilatation [3]
  6 $$\begin{aligned} E_{\mathrm {QC}} (\sigma _1 , \sigma _2 ) = \max \limits \, \left\{ \displaystyle \frac{\sigma _1}{\sigma _2}, \frac{\sigma _2}{\sigma _1}\right\} \,; \end{aligned}$$
- MIPS energy [25, 26]
  7 $$\begin{aligned} E_{\mathrm {MIPS}} (\sigma _1 , \sigma _2 ) = \displaystyle \frac{\sigma _1}{\sigma _2} +\displaystyle \frac{\sigma _2}{\sigma _1} = \displaystyle \frac{\sigma _1^2 + \sigma _2^2}{\sigma _1 \sigma _2}\,; \end{aligned}$$
  Most isometric parametrizations (MIPS) is a quadratic function, widely used for optimizing conformal distortions over triangular domains [26].

**Area distortions:** These distortions estimate dilatation and compression of triangle areas induced by *f*. We use the following measure of the area distortion:

- Unsigned area distortion [27]
  8 $$\begin{aligned} E_{\mathrm {AD}}(\sigma _1,\sigma _2) = \max \limits \, \Bigl \{ |\sigma _1 \sigma _2| , | \, \sigma _1 \sigma _2 |^{-1}\Bigr \}\,; \end{aligned}$$

**Scale distortions:** These distortions assess the degree to which mesh triangles are scaled by *f*. Scale distortions are closely related to discrete harmonic mappings [28] and to stretch minimization mappings. We use the following scale distortions:

- *Dirichlet* energy [25]
  9 $$\begin{aligned} E_{\text {Dirichlet}} \, (\sigma _1 , \sigma _2) = \frac{1}{2}\, \Bigl (\sigma _1^2 + \sigma _2^2\Bigr )\,; \end{aligned}$$
- Conformal factor [21]
  10 $$\begin{aligned} E_{\text {CF}}(\sigma _1,\sigma _2) = \, \displaystyle \frac{\sigma _1 + \sigma _2}{2}\,. \end{aligned}$$
  Note that conformal factors are closely related to conformal distortions such as quasi-conformal dilatation and MIPS energy. Indeed, according to the uniformization theorem [29], any disk topology surface *S* can be mapped into the plane by a conformal map $f_S$. The map $f_S$ can be described by its conformal factors, up to a composition of $f_S$ with a rigid transformation. For this reason, the conformal factor has been used by [21] as a geometric signature for a collection of 3D surfaces.

All of these distortion measures are rotation invariant, since they are functions of signed singular values of the Jacobian. This work aims to show that the dimensionality of the data can be considerably reduced by employing weighted sums of local distortions over different subsets of $\Im$. The obtained quantities will be referred to as a *global distortions*.

### Measuring global distortions

Let *f* be a simplicial map of the mesh $(\mathcal {V},\Im )$, *E* be a local distortion, and $\Im _0$ be a subset of $\Im$. The global distortion of *f*, computed with respect to *E* over $\Im _0$, is then defined as follows:

11 $$\begin{aligned} D_{\Im _0}(f, E) = \frac{\sum \nolimits _{t\in \Im _0} \, E\big (\sigma _1(df_t),\sigma _2(df_t) \big ) \text {area}(t)}{\sum \nolimits _{t \in \Im _0} \, \text {area}(t)}\,, \end{aligned}$$

where $df_t$ is the Jacobian of *f* on *t*, $\sigma _1(df_t)$ and $\sigma _2(df_t)$ are the Jacobian singular values and $\text {area}(t)$ denotes the area of a triangle *t*.

**Fig. 4:**
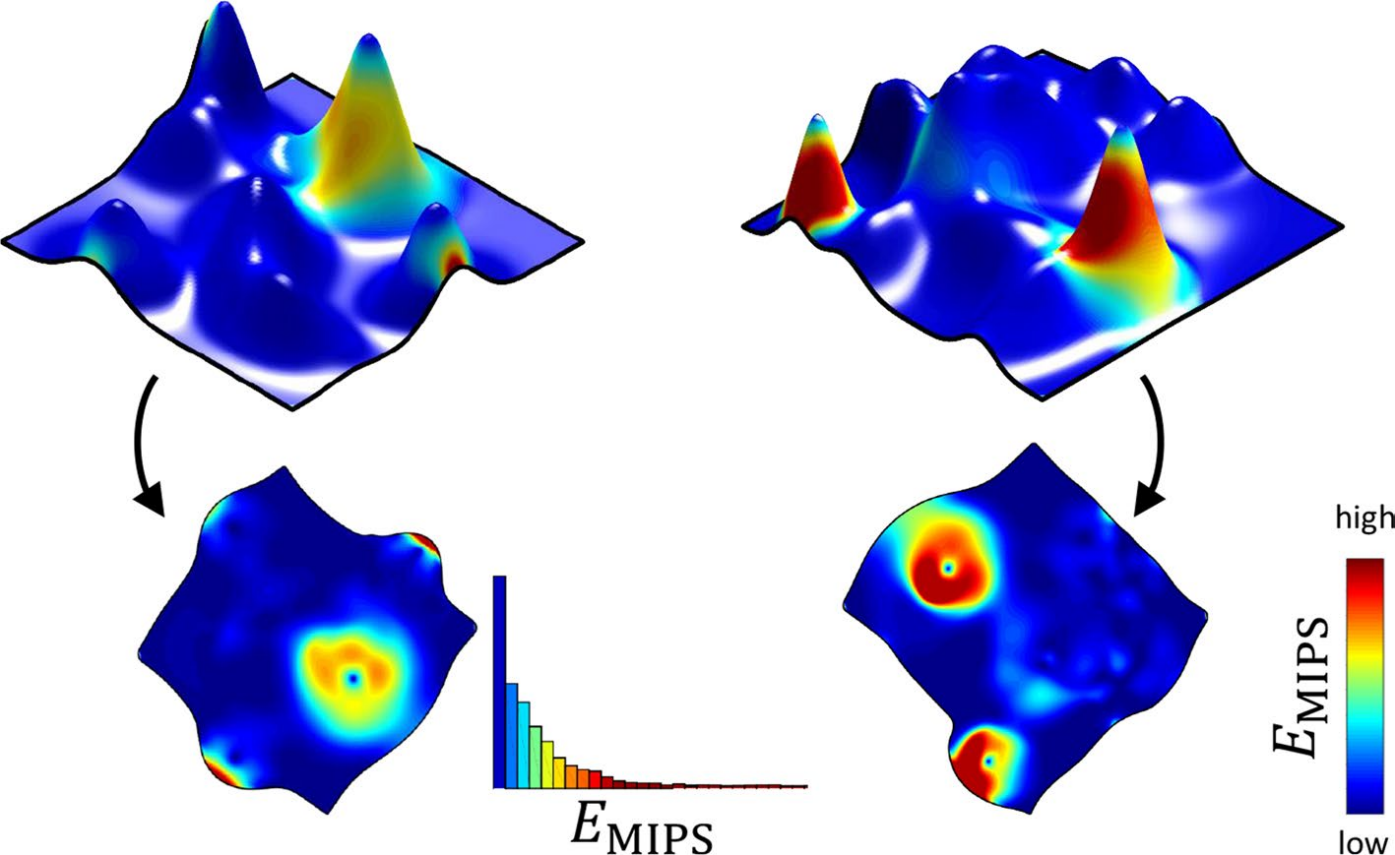
Flattening surfaces and measuring the resultant geometrical distortions, attained as shape descriptors. The process is visualized for the MIPS distortion energy, defined by (7). We first use ABCD algorithm [2], initialized with Tutte embedding, to map the triangulated surfaces into the plane. We then compute the Jacobian singular values $\sigma _1(t)$ and $\sigma _2(t)$ over each mesh triangle *t*. Finally, we compute for each distortion measure $E(\sigma _1,\sigma _2)$ and mapping *f* the two quantities $E_1(f)$ and $E_2(f)$, defined according to (11) and (14)

**Fig. 5:**
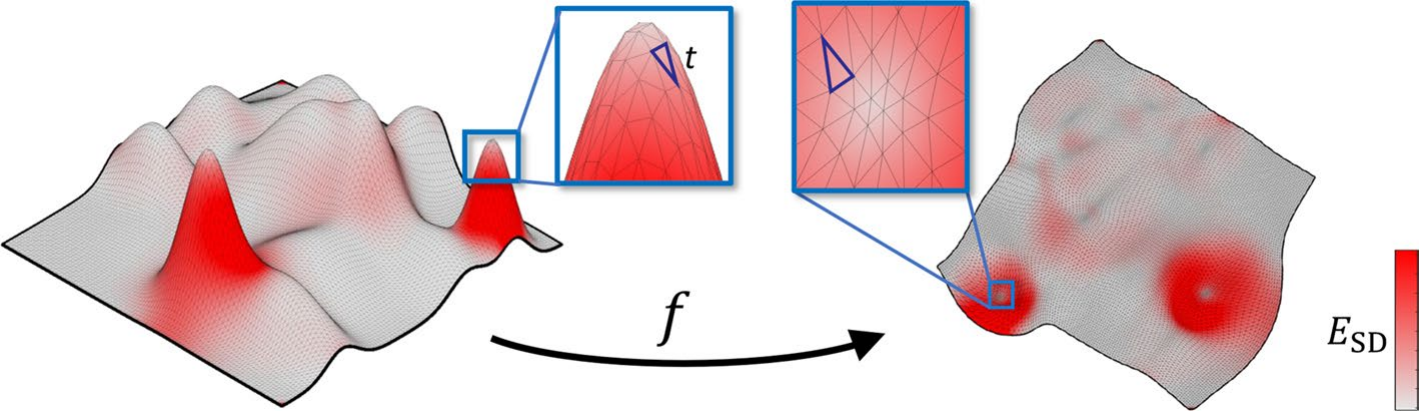
Visualization of a simplicial map *f* with color-encoded local distortions $E_{\text {SD}}$ , defined by (5). We show how an individual triangle *t* is mapped under an affine component $f_t$ of the map *f*

In many cases, values of local distortions are distributed non-uniformly over mesh triangles. As demonstrated by Figs. 4 and 5, a small number of highly distorted triangles may have more impact on the global distortion $D_{\Im }(f, E)$ than the rest of the mesh triangles. Therefore, in order to extract more information from each distortion measure, one can divide the triangle set $\Im$ into a number of disjoint subsets. We employ this approach to extract more features for each distortion measure $E(\sigma _1,\sigma _2)$ and to compensate for the adverse effects of a non-uniform distribution of distortions. In particular, we divide triangles into the two subsets according to *triangle frequency*.

Let’s define $f_{cg}(t)$ the frequency of the center of gravity of triangle *t*, and

12 $$\begin{aligned} \overline{f_{cg}} = \mathrm {median}(f_{cg}(t)),~ t \in \Im \,, \end{aligned}$$

the median over the frequencies of all the triangles of the surface. We then define

13 $$\begin{aligned} \begin{aligned} \Im _1 = \{t | t \in \Im ,~ f_{cg}(t) < \overline{f_{cg}}\}, \\ \Im _2 = \{t | t \in \Im ,~ f_{cg}(t) >= \overline{f_{cg}}\}\,, \end{aligned} \end{aligned}$$

The global distortions are computed for the two subsets of triangles. We will denote these features by $E_1(f)$ and $E_2(f)$, for short. That is,

14 $$\begin{aligned} E_i(f) = D_{\Im _i}(f,E),~ i=1,2\,, \end{aligned}$$

where $D_{\Im _i}$ is defined according to (11).

To summarize, we measure global distortions over the two subsets of triangles and use the obtained quantities as shape descriptors of spectrogram surfaces. This approach has the following advantages over the distortion-based models, previously proposed for shape analysis [17, 21]:

1. A wider set of distortion measures is used.
2. The overall number of features is further increased by dividing distortions into the low and high frequencies.
3. The method operates on triangular meshes instead of tetrahedral meshes. Compared with the volumetric method of [17], extracting features by our algorithm results in a lower computation cost.^2^

## Related work

There exist several approaches to computing shape descriptors for a collection of 3D objects, other than the deformation-based method that we prefer. Among them are:

1. **Spectral methods**, whereby shape descriptors are derived from discrete representations of the Laplace–Beltrami operator, defined on surfaces [30]. Cotangent weights are most commonly used for approximating Laplace–Beltrami operators over meshes. By using cotangent weights, the Laplace operator action on a mesh *M* can be represented by a sparse Laplacian matrix $L=L(M)$. In such a case, the spectral descriptors of $M=(\mathcal {V},\Im )$ are often defined as *n*-largest eigenvalues of *L*, for a constant number $n < |\mathcal {V}|$ [31].
2. **Metric methods**. These methods represent each mesh *M* by a matrix *G* of pairwise distances between vertices of *M*. Usually, these are the Euclidean or geodesic distances. A dissimilarity measure between two meshes $M_1$ and $M_2$ is defined in the metric approaches as a function of the distance matrices $G_1$ and $G_2$ of these two meshes. For example, metric descriptors of triangulated surfaces can be obtained by solving the problem of the general multi-dimensional scaling (GMDS) [32], or by solving other related problems that involve computations of geodesic distances [33, 34].

Global changes of geometric structures have been also studied in the context of medical images [11].

Furthermore, the problem of flattening triangular meshes into the plane, also referred to as the parametrization problem, constitutes one of the central issues in geometry processing. Consequently, there exist many algorithms for flattening triangulated surfaces [35]. These algorithms are aimed at computing a locally injective parametrization that minimizes distortions of fundamental geometric quantities, such as angles and lengths.

## Experiments

The first experiment reported is concerned with detecting respiratory pathologies by analyzing lung sounds. There exist several deep learning- and model-based methods for automatic classification of lung pathologies based on their fingerprints that are hidden in the pulmonary sounds. For instance, the recent method of [36] implements a deep transfer learning-based multi-class classifier for diagnosis of COVID-19, using cough recordings. Chanbres et al. [37] employ the algorithm of the Essential library [38] for extracting sound features from cough recordings. This system was trained on the dataset of the ICBHI 2017 challenge [39] by using a boosted decisional tree algorithm to classify sounds like crackles and wheezes.

For the second use-case, we selected accent detection from speech sounds. Hossain et al. [40] have used the MFCC features and then applied classical machine learning classifiers (k-nearest neighbors and support vector machine) to detect the accent. Another study [41] used a convolutional neural network directly on the raw speech. They trained their model on Wildcat Corpus of Native and Foreign-Accented English [42] and got an accuracy of 88%.

### Lung sounds

#### Database

The Respiratory Sound Database [39] was used for the first experimental implementation of our approach. A total of 918 lung sounds recordings from 126 patients were used. This database incorporates seven different pathologies: URTI, Asthma, COPD, LRTI, Bronchiectasis, Pneumonia, Bronchiolitis, and healthy recordings.

**Fig. 6:**
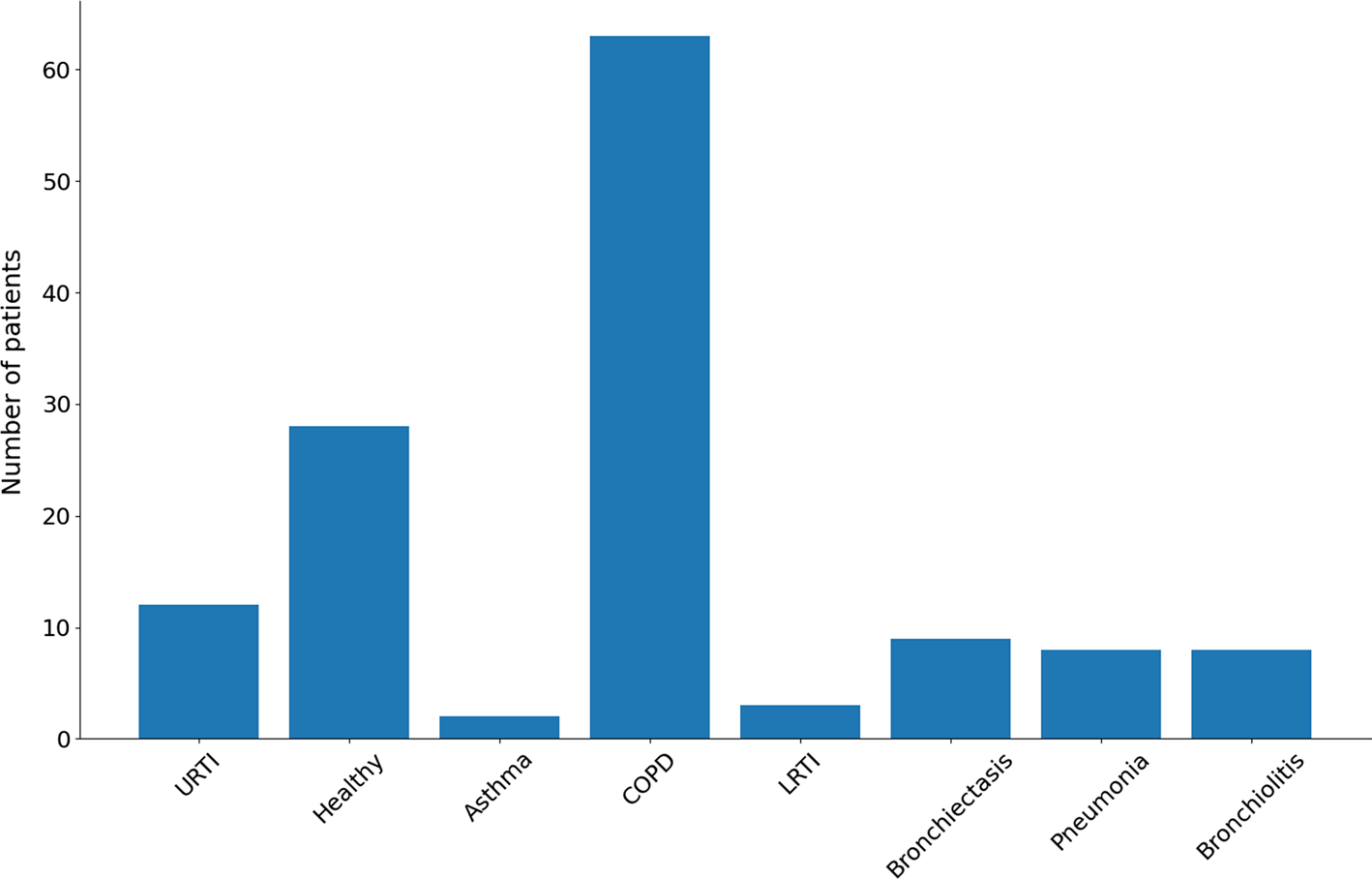
Distribution of the pathological and healthy cases included in the database. The database is imbalanced. About 50% of the patients have COPD disease

The histogram depicted in Fig. 6 presents the distribution of the pathologies among the cases included in the database. Due to the very low occurrence of the Asthma and LRTI pathologies, the corresponding recordings were excluded.

#### Preprocessing

One of the major problems that one must overcome in the process of analysis of lung sounds is the low *S*/*N* level. Sounds generated by instruments and other ambient activities affect significantly the quality of the lung sound signal. It is therefore crucial to improve the level of the *S*/*N* without distorting the stethoscope’s signal.

Our algorithm employs the classical Savitzky–Golay filter [43] for denoising lung sounds. The purpose of this filter is to smooth the signal and improve the SNR without altering the desired lung sounds signal. This filter has been widely used in the field of time series analysis [44], especially for lung sound analysis [45]. The filter aims to fit a specific polynomial suitable for a signal frame, using least squares method. The central point of the window is replaced with that of the polynomial, producing a smoother signal.

Denote a polynomial of the degree *N* by

15 $$\begin{aligned} p(n) = \,\sum \limits _{k=1}^N\, a_k n^k \, , \end{aligned}$$

then, the aim of Savitzky–Golay filter is to minimize the following error:

16 $$\begin{aligned} \mathcal {E}_N = \,\sum \limits _{i=-M}^M\, \Bigl (p(i) - x[i])^2\Bigr ) \,, \end{aligned}$$

where $2M+1$ is the width of the window and *x*[*i*] is the corresponding sample of the signal.

**Fig. 7:**
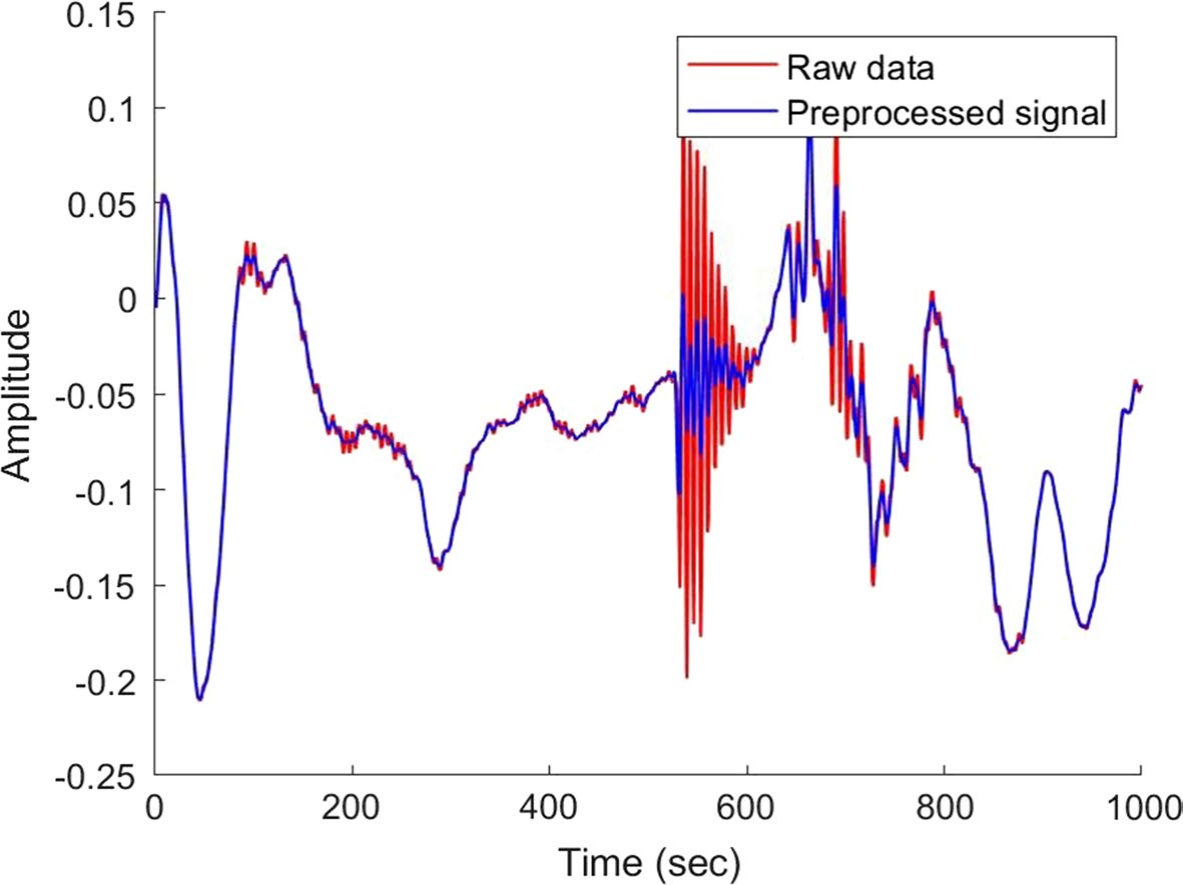
Effect of the Savitzky–Golay filter on lung sounds recording. The signal is very noisy; over the segment of the time period 530–580 s, the Savitzky–Golay filter eliminates the sharp spurious changes

A large value of *M* will yield a smoother signal, but it may neglect some important variations in the signal. A low value of *M* may ‘over fit’ the data. Secondly, *N*, which specifies the degree of the polynomial may produce a smooth signal for low values. On the other hand, high value of *N* may ‘over fit’ the data. By experimenting with various combinations of these filter parameters, we converged on the values of $N=3, M=11$ that yielded the best results. Figure 7 shows an example of a filtered signal, superimposed on the corresponding raw data.

#### Implementation

Two examples of classification tasks are presented: a multi-class classification, incorporating five pathologies and healthy recordings, and a binary classification. Each of the five pathologies is presented against the class of healthy recordings.

The dataset was subdivided into training set (80%) and test set (20%). For each task, several classifiers were experimented with: logistic regression (LR), support vector machine (SVM), random forest (RF), *K* nearest neighbors (KNN), AdaBoost (AB), and XgBoost (Xb). For all of these models, we used 16 engineered features. For each model, hyper-parameters such as the number of estimators or number of neighbors were optimized using fivefold cross-validation. A large random grid of hyper-parameters was searched for. In the case of the multi-class classification, the performance measure used for optimization was the accuracy, whereas for the binary tasks the area under the receiver operating characteristics curve (AUROC) was used. A weight has been assigned to each class, inversely proportional to the class frequencies in the training set.

Training examples were divided into training and validation set, for each iteration of the cross-fold, by stratifying among patients, which means that several recordings from the same patient are always included in the same set. All the models were trained on the same test set. That is, for all the models, the database was split into the same training and test subsets.

The following metrics were used for the performance evaluation:

17 $$\begin{aligned} \mathrm {Accuracy}= & {} \frac{\mathrm {TP} + \mathrm {TN}}{\mathrm {TP} + \mathrm {TN} + \mathrm {FP} + \mathrm {FN}}, \end{aligned}$$

18 $$\begin{aligned} \mathrm {Recall} = \frac{\mathrm {TP}}{\mathrm {TP} + \mathrm {FN}}, \end{aligned}$$

19 $$\begin{aligned} \mathrm {Jaccard}= & {} \frac{\mathrm {TP} + \mathrm {TN}}{2(\mathrm {P}+\mathrm {N}) - (\mathrm {TP} + \mathrm {TN})}, \end{aligned}$$

where TP, TN, FP, and FN are the true positives, true negatives, false positives, and false negatives , respectively. P denotes the number of positive samples, and N is the number of negative samples. The area under the ROC curve, AUROC, is computed.

#### Baseline

A baseline (i.e., a reference mode) has to be created for comparison of the model created with. A different approach has been selected for this purpose, based on a set of features that have been handcrafted. Twelve mel-frequency cepstrum coefficients (MFCCs) were extracted from the audio files: MFCC is the most widely used feature extraction method in automatic speech recognition [46]. In the feature extraction phase, six statistical parameters have been extracted from each of the 12 MFCC coefficients as follows: mean, standard deviation, min, max, mean of the absolute difference, and standard deviation of the absolute difference, altogether 72 features.

The reference model has been applied to all classifiers, with the same training process as for the proposed model. The models that we use for comparison have been trained and tested on the same train/test subdivision of the data.

Finally, the MFCC-based model has been combined with the proposed model (based on distortion measures). For each recording, 88 features have been computed: 16 features based on distortion measures (2 for each distortion) and 72 features based on MFCC coefficients. As the number of features increased significantly, a feature selection step has been applied, based on the ranking of features, determined by implementation in the random forest classifier. Altogether, 45 features have been selected.

A second baseline has been created, to benchmark the proposed method, wherein the model adopted from Fraiwan et al. [47] was implemented. It is in essence a combination of 1D convolutional neural network and a bidirectional long short-term memory.

#### Results

**Fig. 8:**
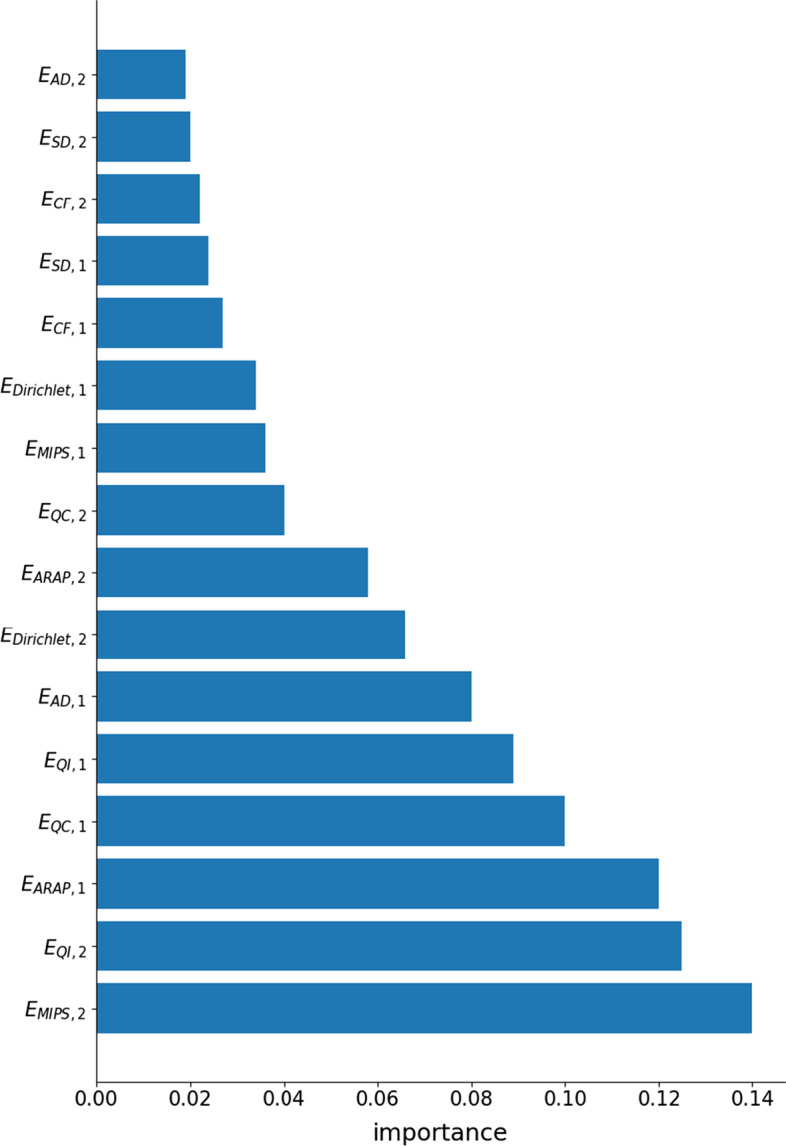
Ranking according to feature importance, determined by using a random forest classifier. The ranking provides insight into the most useful features in the classification process

The results obtained by the different models are summarized in Table 1 for the multi-class classification task, and in Table 2 for the binary task. Ranking of the features according to their importance, as determined by random forest classifier, is depicted in Fig. 8.

**Table 1:** Results obtained by means of the different models in the multi-class task of classifying lung sounds

	Accuracy	Recall	Jaccard
MFCC	0.76	0.80	0.69
Fraiwan	0.85	0.86	0.78
Our model	0.88	0.89	0.80
Combined	0.90	0.91	0.88

The best score for each metric is underlined

**Table 2:** AUROC obtained by means of the different models in the binary classification for each pathology

	URTI	COPD	Bronchiectasis	Pneumonia	Bronchiolitis
MFCC	0.82	0.98	0.95	0.90	0.81
Fraiwan	0.87	0.99	0.94	0.88	0.94
Our model	0.89	0.99	1.00	0.87	1.00
Combined	0.93	1.00	1.00	1.00	1.00

The best score for each pathology is underlined

The proposed model obtains a better AUROC than the baseline models for almost all the binary tasks. For differentiation of pneumonia pathology from the rest of diseases, the proposed model yields a lower AUROC value than the two baseline models (0.87 for our model, versus 0.90 for MFCC and 0.88 for Fraiwan).

**Fig. 9:**
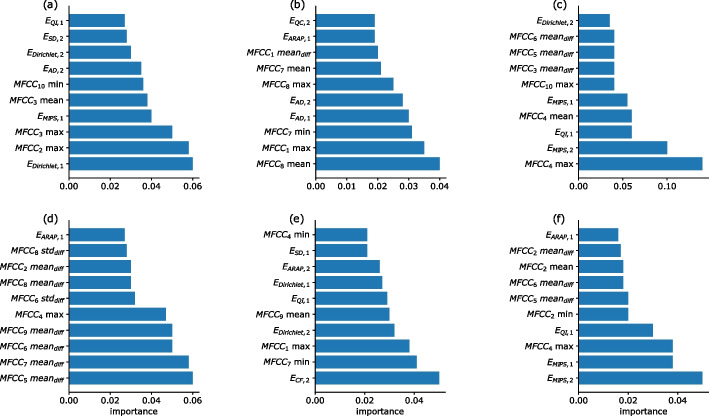
Ranking efficacy of the 10 best features of the combined model, determined by means of the performance of the random forest classifier. **a** The ranking is presented for the binary task of identifying bronchiectasis; bronchiolitis (**b**); COPD (**c**); pneumonia (**d**); URTI (**e**). Finally (**f**) presents the ranking for the multi-class task of classifying the five pathologies

Figure 9 presents the ranking of the features, for each of the five binary tasks and the multi-class task of identifying the five pathologies. Although there are 16 distortion measures features and 72 MFCC features, for most of the pathologies the occurrence of the distortion measure features is relatively high. In particular, there are six distortion measures out of ten most highly ranked features for the bronchiectasis and URTI pathologies. Likewise, distortion measures appear among the four most highly ranked features used in classification of the bronchiolitis and COPD diseases. Indeed, for this pathology the MFCC-based model outperformed the proposed model. However, in the case of identifying the pneumonia, then only a single distortion measure appears in the feature ranking list. Indeed, for this pathology the MFCC-based model outperformed the proposed hybrid model.

### Speech sounds

#### Database

The L2-Arctic database [48] was used for this analysis. This is a speech corpus of non-native English speakers. It contains 24 different speakers, whose first language is one of the following: Hindi, Korean, Mandarin, Spanish, Arabic, or Vietnamese. The database includes both male and female speakers for each accent. Each speaker was recorded for approximately one hour of read speech. The task of accent detection was applied.

#### Preprocessing

The maximal overlap discrete wavelet transform (MODWT) [49, 50] was applied. This transform uses a combination of high-pass and low-pass filters to decompose the sequence. The threshold function proposed by [51] was adopted.

In the case of speech sound, a mel spectrogram was applied, instead of the classical STFT. Parameters of the mel spectrogram, such as number of mel coefficients, type of window and its length, were chosen by cross-validation.

#### Implementation

A multi-class classification task of detecting the accent was performed on the six available accents. The dataset was divided into $80\%$ training set and $20\%$ test set according to speakers. The same classifiers were used as in the following experiment, along with the same training process.

#### Baseline

The model of Jiao et al. [52] was implemented as a baseline for this experiment. The model was tested on the INTERSPEECH 16 Native Language Sub-Challenge, which contains one speech sample from 5132 speakers and yielded an accuracy of $50.2\%$, with 11 different accents. The model was composed of two parallel networks: a DNN which analyzes long-term features and a RNN which analyzes short-term features from frames of the speech signal. The final decision was determined by a probabilistic fusion algorithm.

#### Results

**Fig. 10:**
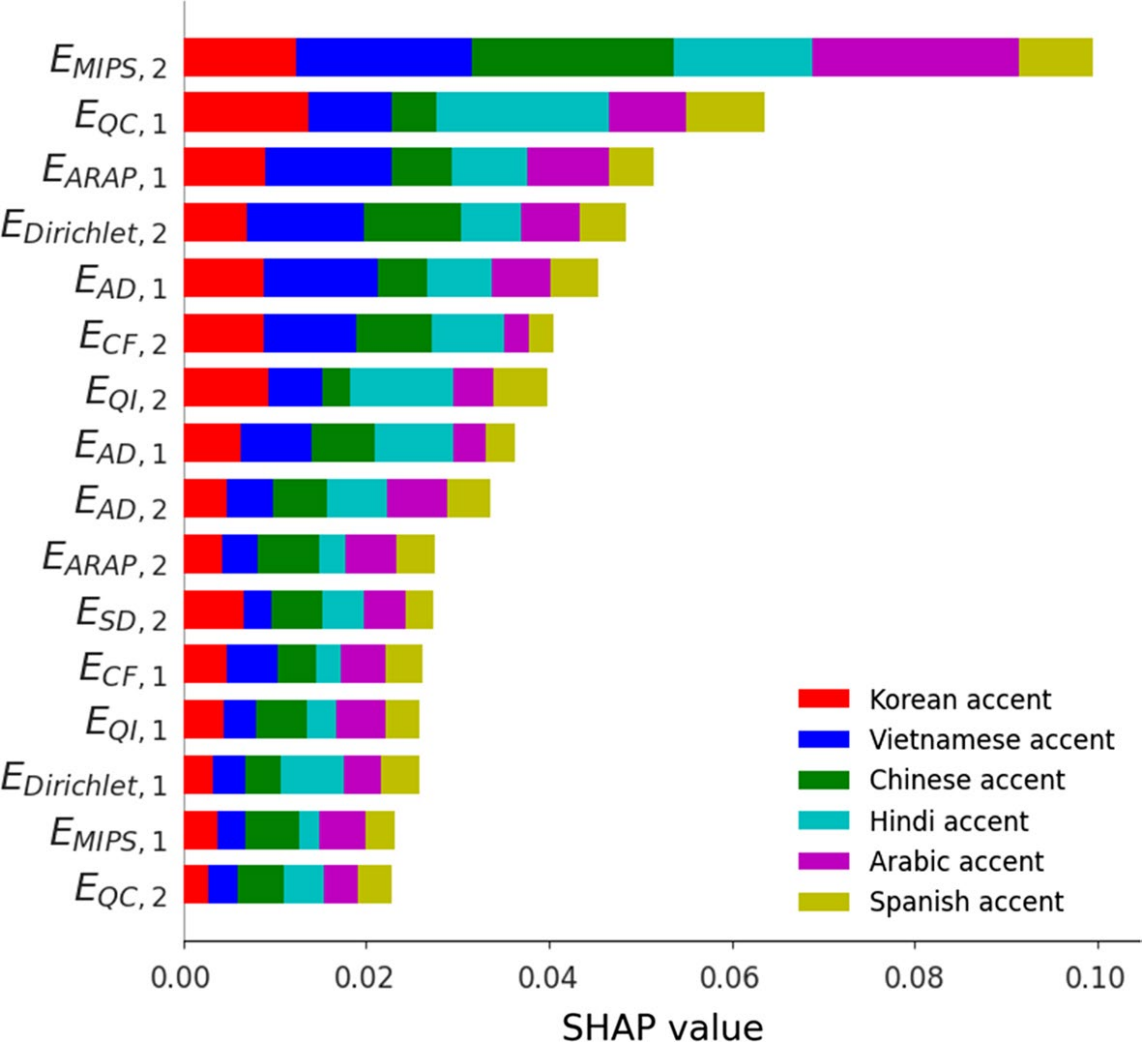
Ranking of the distortions measures according to their SHAP values, per class

The results obtained with the different models are summarized in Table 3, while Fig. 10 presents the ranking of the features according to SHapley Additive exPlanations (SHAP) values [53, 54]. These values allow to interpret the global model structure using local explanations. An important observation is that some features repeat among the top 10 in both experiments (Figs.  8 and 10), e.g., $E_{\mathrm{MIPS}, 2}$, $E_{\mathrm{Dirichlet}, 2}$, and $E_{\mathrm{CF}, 2}$. To summarize, the proposed model outperformed the baseline models under all the measured metrics.

**Table 3:** Results of the multi-class classification

	Accuracy	Recall	Jaccard
Jiao	0.58	0.62	0.62
Our	0.72	0.73	0.75

## Discussion

The purpose of our present paper is to present our new geometric approach to signal representation, analysis, and similarity assessment in the context of 1D signals, using as examples the applications to lung sounds and speech signals, rather than to establish a benchmark for a specific signal by using impressively large dataset(s). We have applied our algorithm to signals with well-defined structure, which lends itself to representation of 2D manifold embedded in a 3D Euclidean space, endowed with extrinsic well-defined geometric structure. These examples represent a wide range of important applications. Signals with less defined structures, or even containing singularities, may have to be conditioned by reproducing kernels [10, 55] in order to be represented by structured manifolds and thereby exploit our geometric approach. The successful results obtained so far by the application of our novel approach to classification, based on distortion measures, highlight a possible interesting extension of the present work which we intend to pursue by considering higher-dimensional distortion measures, such as those that may be applied to complex spectrograms [56]. A spectrogram surface can be represented by the tetrahedral volume enclosed by the surface and the plane $z=0$. In such a case, sound spectrograms can be characterized by 3D distortions induced by mapping tetrahedral meshes into canonical domains. Although the tetrahedral approach entails a higher computational complexity, the extra computational cost should be justified by obtaining, more accurate results due to the fact that volumetric distortions can detect both of the changes that are imposed on the boundary surface and the changes made in the interior volume.

In this paper, well-constrained signals have been selected to demonstrate the added value of the method proposed. Indeed, speech and lung sounds are well-structured signals, which result in well-defined geometric structures. Nevertheless, the stability of the algorithm has been thoroughly studied. First, the geometric component of the proposed method, ABCD, is designed to be noise resistant and robust to inverted and collapsed triangles, as it has been confirmed by various tests conducted by Naitsat et al. [2]. We refer readers to [57] for theoretical analysis of the distortion minimization stability. In particular, the study of [57] has analyzed how stable is a local optimization of various distortion measures under noise perturbations. Further, these conclusions were used in [2] to propose a local–global optimization scheme for ABCD algorithm. The local/global optimization approach is designed to be both noise resistant and fast converging.

Regarding the important issue of the stability of our algorithm, with reference to the distortions used in this study, it should be noted that in general, most of distortions used in our paper can be divided into the two main categories: the so-called barrier distortions and non-barrier distortions. The former, such as $E_\mathrm{SD}$ and $E_\mathrm{QI}$, are equal to their global minima for rigid transformations and diverge to infinity when singular values approach zero. In [57], we have analyzed these properties and concluded that minimizing barrier distortions is numerically stable under an injective initialization. In a later study, Naitsat et al. [2] introduced the ABCD algorithm to deal with non-injective initializations while maintaining numerical stability. Non-barrier distortions, such as $E_\mathrm{Dirichlet}$, are bounded from above and thus are less insensitive to noise. Furthermore, according to [1], many non-barrier distortions are convex with respect to vertex coordinates. Minimizing these measures is stable and fast converging according to the convex optimization theory.

Moreover, the way we employ Delaunay triangulation has no adverse effects on the algorithm stability. Signals are sampled on the same 2D time–frequency grid. We then connect these grid points via Delaunay triangulation to get a planar mesh. Finally, we extend the coordinates of planar mesh vertices by adding spectrogram values as vertex heights. Thus, we use triangle meshes with the same connectivity and different vertex coordinates to represent 1D signals, limiting any potential instability that may be introduced by the Delaunay algorithm. To deal with variations in triangulation of not-well-constrained signals, we can use the following property: According to multi-resolution analysis of distortion measures [1], we can first minimize distortion induced by mapping of coarse triangulated domains and then subdivide source and target domains for obtaining a low distortion map in a higher resolution, without degrading the results. Since subdivisions reduce variations in triangle shapes and do not increase distortion measures, subdivisions can be used to induce regular meshes with similar structure for representing non-uniformly sampled signals. The robustness of the proposed algorithm has been tested experimentally (Additional file 1: Sect. 7.2), indicating that the algorithm is robust up to a level of standard deviation of 50% of the lung sounds signal, and up to an SNR of − 20 dB of the speech signal.

It should be interesting to combine our model with various types of shape descriptors, such as the metric and spectral geometrical features, listed in Sect. 2.2. It is likewise interesting to examine more methods for discretizing the surface (or a higher-dimensional manifold) representation. Indeed, the choice of the triangulation method (in this study Delaunay) has a profound effect on the results. An ideal triangulation, composed of only equilateral triangles of equal size, could improve significantly the results. In particular, a curvature-based method [10] can be used for a more accurate sampling of spectrogram images and for constructing triangular meshes with an optimal number of vertices. This can be done by viewing the spectrograms computed as two-dimensional Riemannian manifolds. Furthermore, the phase of the spectrogram should be incorporated into the discretization process. As proven in [58], the operator mapping a function to its spectrogram samples on a lattice is not injective. Several recent studies have incorporated the phase in their work on spectrogram (e.g., [59, 60]). Although in this paper we compare meshes by mapping them into the plane, our algorithm can be extended to a more general setting. In particular, the obtained meshes could be compared pairwise, or each mesh could be compared to a subset of geometric domains that represent different classes of input signals. For example, in the case of speaker identification, we can compute a mean shape $S_i$ for each of the reference speakers $i=1,\ldots N$ and then compare geometric distortions induced by mapping spectrogram surfaces onto the obtained mean shapes $S_1,\ldots ,S_N$.

Finally, we stress that our approach to the classification of one-dimensional signals is also applicable to higher-dimensional signals. Distortion measures can be extended in a straightforward manner to $\mathbb {R}^n$ and to piecewise linear manifolds embedded in $\mathbb {R}^n$, for any $n\ge 2$. Indeed, if $\varvec{f}:\mathbb {R}^n\rightarrow \mathbb {R}^n$ is a simplicial map and *s* is an *n*-dimensional simplex, then a local distortion of $\varvec{f}$ over *s* can be expressed by a function $E(\sigma _1,\sigma _2,\dots ,\sigma _n)$, where $\sigma _i$ denotes the $i{\text {th}}$ singular value of the Jacobian matrix $d\varvec{f}_s\in \mathbb {R}^{n \times n}$. So that our distortion-based analysis of surfaces is extended to *m*-manifolds embedded in $\mathbb {R}^n$ and to their discrete representations, for any $2 \le m\le n$. For instance, consider a simultaneous recording of different time-varying signals such as pulmonary sounds, heart rate, oxygen saturation, and body plethysmography. Instead of computing a surface representation for each signal separately, one can represent a *n*-channel data stream by a 2-manifold embedded in $\mathbb {R}^n$. The obtained manifold can be discretized using the sampling method of [10] and a Delaunay-based algorithm for triangulation. An extension of our approach to higher-dimensional manifolds thereby allows a more general analysis of multichannel biomedical (or other) sets of data, collected from various devices. We therefore consider other applications of the proposed distortion-based model in related fields of biomedical signal processing, medical imaging, and voice recognition.

## Supplementary Information

**Additional file 1.** Computational cost and robustness of the algorithm proposed.

## Data Availability

The datasets generated during and/or analyzed during the current study are available at [61, 62].

## Footnotes

1 In the context of the representation on the surface of the local spectrum, the geometric distortions assess how much the local spectrum of the sound is affected by the simplicial mapping *f*.

2 We use triangular meshes because our data is represented by disk topology surfaces. However, it is possible to represent this data by tetrahedral meshes and to estimate volumetric distortions of these meshes. [See Sect.5 for more details.].
