# Supplementary material for: Classification of audio signals using spectrogram surfaces and extrinsic distortion measures
Source: EURASIP J Adv Signal Process. 2022 Oct 22;2022(1):100. doi: 10.1186/s13634-022-00933-9 (PMC9589786; doi:10.1186/s13634-022-00933-9)
Supplement: Supplementary file 1 — Additional file 1. Computational cost and robustness of the algorithm proposed. [file 13634_2022_933_MOESM1_ESM.pdf]

7. Supplements

7.1. *Computational cost*

The runtime for extracting the distortion measures was 7.87 ± 1.02 seconds per sample, in the case of the first experiment. For the second experiment, it was 8.21 ± 0.29 seconds per sample. The process of feature extraction was implemented in parallel on 40 cores, resulting in a runtime of 182 seconds for the first experiment, and 592 seconds for the second experiment. The peak memory usage during the extraction of distortion features was 54.4 Gb for the first experiment, and 62.0 Gb for the second. For comparison, the Fraiwan baseline had a peak memory usage of 84.3 Gb, and for Jiao baseline it was 94.1 Gb. Once the features were extracted, the Bayesian search for hyper-parameter optimisation was ran for 30 minutes per experiment.

The experiments were ran on a NVIDIA DGX station, comprised of 40 CPUs (Intel(R) Xeon(R) CPU E5-2698 v4), with a total of 264 Gb of ram.

7.2. *Robustness of the algorithm*

To assess the robustness of the algorithm with reference to noise-like perturbations, additive Gaussian noise was applied to the signal. For a noise at level x, a Gaussian noise with variance $\sigma = x * signal$ is generated, where *signal* is the original signal studied. As shown in Fig. S1, when the level of the noise decreases, the distortion measures converge to the original values of the noiseless signal. Panel (a) shows the mean and interquartile error of the distortion measures, with respect to the noiseless signal. Panel (b) shows the classification error of the final model, due to the contaminated noise. Up to 30% of noise, the error of the distortion measures was of less than 1%, and up to 50% of noise, the classification error was of less than 1%.

A second experiment has been run, using the speech sounds. Real room impulse responses and isotropic noise [63] has been added to the signals, with different SNR levels. As shown in Fig. S2, the algorithm is robust up to an SNR of -2 dB.

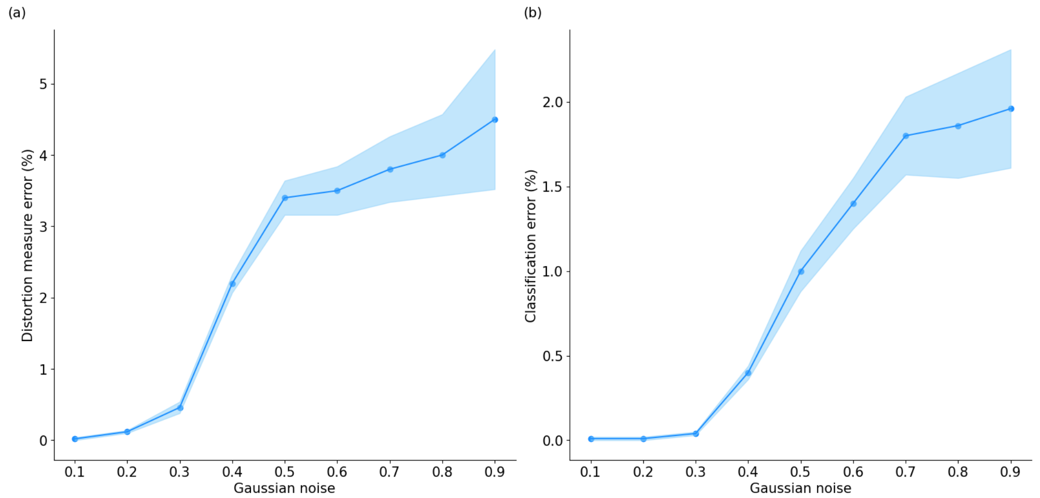

Figure S1: On panel (a), mean and interquartile error of the distortion measures, with respect to the noiseless signal. On panel (b), mean and interquartile of the classification error of the noised signals.

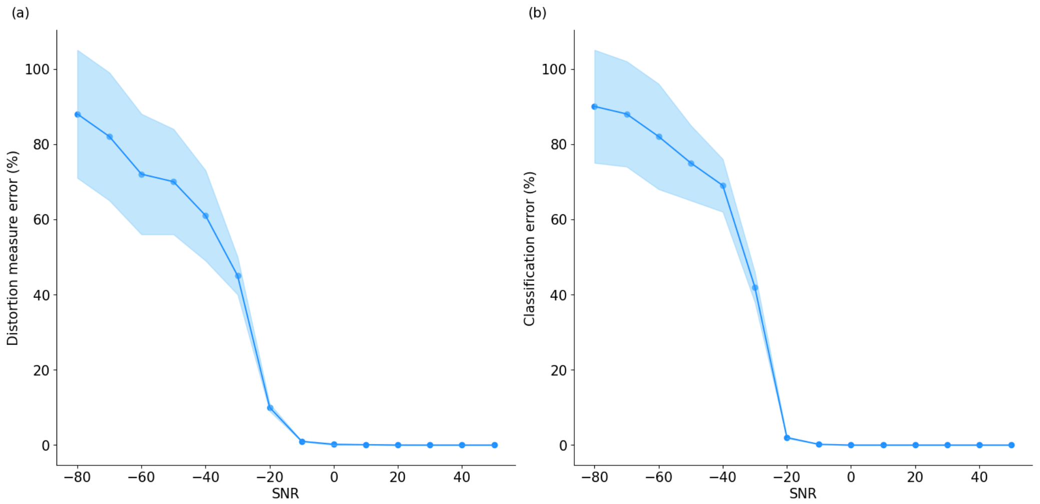

Figure S2: On panel (a), mean and interquartile error of the distortion measures, with respect to the noiseless signal. On panel (b), mean and interquartile of the classification error of the noised signals.
